# The Effect of Preoperative Anxiety on Motor and Sensory Block Duration and Effectiveness in Spinal Anesthesia

**DOI:** 10.1155/2024/8827780

**Published:** 2024-08-19

**Authors:** Yadigar Yılmaz, Esra Durmayuksel, Tuna Erturk, Ferda Yılmaz Inal, Dilek Metin Yamac, Aysin Ersoy

**Affiliations:** ^1^ Sultan 2. Abdulhamid Han Education and Research Hospital Department of Anesthesiology and Reanimation, Istanbul 34668, Türkiye; ^2^ Bahçeşehir University Faculty of Health Sciences Nursing Department, Istanbul, Türkiye; ^3^ Istanbul Medeniyet University Faculty of Medicine Department of Anesthesiology and Reanimation 34722, Istanbul, Türkiye

## Abstract

**Introduction:**

The aim was to evaluate the effect of preoperative anxiety on the sympathetic block that developed after spinal anesthesia and therefore the duration of motor and sensory blockade.

**Materials and Methods:**

After the approval of the ethics committee, 90 patients between the ages of 18 and 55 years who were to be operated under spinal anesthesia were included in the study. Preoperative anxiety of the patients was evaluated with the Spielberger trait and State Anxiety Scale and Visual Analog Scale (VAS). The Bromage scores of the patients were followed up intermittently. Onset time of sensory block, onset time of motor block, and motor block recovery time were recorded. Cases with bradycardia and hypotension were noted.

**Results:**

No statistically significant correlation was found between the duration of motor block onset (5.81 ± 4 min), the sensory block onset time (0.89 ± 0.4 min), and the motor block recovery time (92.06 ± 36.9 min) with other variables. VAS (5.81 ± 2.5), STAI-1 (40.4 ± 9.8), and STAI-2 (41.69 ± 8.2) values had a statistically significant effect on the occurrence of bradycardia (14.4%). The variables of VAS, STAI-1, STAI-2, sensory block onset, motor block onset, and motor block recovery time were statistically significantly higher in women (mean 5.24 ± 2.4, 38.97 ± 9.9, 41.43 ± 8.7, 0.89 ± 0.42, 5.64 ± 3.82, and 88.77 ± 38.74 in males and mean 7.15 ± 2.1, 43.74 ± 8.9, 42.30 ± 7.0, 0.88 ± 0.27, 6.20 ± 4.35, and 99.70 ± 31.70 in females, respectively).

**Conclusion:**

It was observed that preoperative anxiety had no effect on motor and sensory block onset and duration.

## 1. Introduction

Preoperative anxiety is encountered in 60–80% of patients with planned surgical operation and adversely affects the surgical operation, anesthesia, and postoperative recovery [1, 2]. Worries about surgery, fear of the unknown, general concerns such as being away from home and relatives, loss of control, missing social life, and anesthesia-related concerns such as not being able to wake up after surgery, feeling pain, waking up during surgery, and death are the main causes of preoperative anxiety.

Anxiety is accompanied by stimulation of the sympathetic nervous system. It may cause intraoperative tachycardia, hypertension, arrhythmia development, impaired pain perception, increased need for anesthetic drugs, and various complications (nausea, vomiting, fatigue, tachycardia, and respiratory system problems) [3–5]. It may negatively affect recovery, patient satisfaction, and quality of life by prolonging the hospital stay of the patient [3, 5].

Spinal anesthesia is a simple and reliable technique with a success rate of over 90% and low cost [6]. However, prolonged motor blockade may extend the time spent in hospital [7]. About one-third of patients who have surgery under regional anesthesia are very anxious before surgery [4]. It is known that spinal anesthesia causes hypotension by blocking sympathetic efferent neurons. It was shown that more significant hypotension develops after spinal anesthesia in patients with higher sympathetic activation at baseline [8]. It may be hypothesized that the sympathetic activation caused by anxiety [8, 9] may make the symptoms due to sympathetic blockade in spinal anesthesia more pronounced.

The aim of this study was to evaluate the effect of preoperative anxiety on the sympathetic block developing after spinal anesthesia and on the duration of motor and sensory blockade.

## 2. Materials and Methods

This prospective observational study was performed after ethical committee (Okmeydani Training and Research Hospital Ethics Committee, 05.03.2019/48670771-514.10) approval, and written informed consent was obtained from all participants. The study was performed with 90 patients aged between 18 and 55 years, with normal BMI and ASA 1-2, who did not have any contraindications for spinal anesthesia, scheduled for elective surgery with spinal anesthesia and surgical interventions that did not interfere with spinal anesthesia.

Those with previous neurological and psychiatric disease symptoms (TIA, syncope, dementia, depression, and bipolar disorder) and drug use, who were allergic to drugs planned for use, undergoing major surgery where a lot of blood loss is expected, who did not want spinal anesthesia, and with contraindications for spinal anesthesia were not included in the study.

All patients were asked to fill in the Spielberger trait and State Anxiety Scale forms on the day of the operation [10]. The patient's current stress was measured with the Visual Analog Scale (VAS). The obtained data were recorded. Premedication was not applied. The patients were taken to the operation room and noninvasive monitoring (heart rate, blood pressure arterial, and pulse oximetry) was done. Preoperatively, patient demographic information, blood pressure, heart rate, and peripheral oxygen saturation values were measured and recorded. Standard monitoring (ECG, noninvasive TA, and pulse oximetry) was done on the patients who were taken to the operating table. Systolic and diastolic blood pressures and heart rates were measured and recorded. Preparations for switching to general anesthesia at any time were made.

The patients were administered 0.03 mg/kg i.v. midazolam on the operating table. After sterilization of the injection area and draping, 4 ml (20 mg) hyperbaric 0.5% bupivacaine (Marcaine heavy) was administered to the patients on the operating table, after entering the subarachnoid space at the L3-L4 or L4-L5 distance with a 25 G Quincke spinal needle and observing cerebrospinal fluid (CSF) flow. The patients were placed in the supine position, and their sensory block levels were checked intermittently and the time interval from intrathecal injection until the patient felt no pain was accepted as the onset of the sensory block. The time interval until the Bromage score was 2-3 after intrathecal injection was accepted as the onset time of the motor block [11]. The operation was started after sufficient sensory and motor blocks developed in the patients. Heart rate (HR), systolic-diastolic-mean blood pressure (BP S/D/M), peripheral oxygen saturation (SpO_2_), and respiratory rate were recorded intermittently in the preoperative and intraoperative periods. Hydration (systolic 80–90 mmHg) and ephedrine i.v. (5 mg, below systolic 80 mmHg) were administered due to hypotension during the operation, and atropine i.v. was administered when bradycardia (pulse rate below 50/min) developed. Cases with bradycardia and hypotension were noted.

Sedation (midazolam) was applied in cases where the patient's anxiety increased and cooperation decreased. The study was terminated when the general condition of the patient deteriorated or the need for general anesthesia developed or spinal anesthesia was unsuccessful. The total duration of the procedure and the total amount of sedation consumed during this time were recorded. If the hemodynamic data were suitable, patients who completed the procedure were taken to the recovery unit for postoperative follow-up. The time of postoperative Bromage scale 0 and possible complications (such as nausea, vomiting, and headache) were recorded. The study was terminated in cases where severe hypotension and bradycardia developed during the measurements, serious drug allergy, or any complications related to the surgical procedure (perforation and bleeding).

### 2.1. Statistical Analysis

The obtained data were analysed using SPSS 20.0 computer program. Analysis of normal distribution for the data was done with the Shapiro–Wilk test. Accordingly, the comparison of the VAS score based on gender was done with the Mann–Whitney *U* test, and the comparisons of STAI-1 and STAI-2 were made with the independent samples *t*-test. The accuracy of the survey results in the groups was calculated with correlation coefficients and statistically significant Pearson test.

## 3. Results and Discussion

### 3.1. Results

A total of ninety patients who were planned to have surgery under spinal anesthesia participated in our study (Table 1). Of these, 78.9% (71) were ASA 1 and 21.1% (19) were ASA 2. The mean durations of the motor block, sensory block onset time, and recovery time of the motor block were 5.81 ± 4, 0.89 ± 0.4, and 92.06 ± 36.9, respectively. When the relationship of all values with the motor block onset time, the sensory block onset time, and the motor block recovery time is examined, a statistically significant, positive, moderate relationship was found between the duration of recovery of the motor block and the duration of the operation (Pearson correlation analysis; *r* = 0.530; *p* < 0.001). Apart from this, no statistically significant relationship was found between the duration of motor block onset, sensory block onset, and motor block recovery and other variables (Pearson correlation analysis; *p* > 0.05) (Table 2).

Bradycardia developed in 14.4% (13) of the patients, and atropine was administered. In 16 patients, hypotension developed and ephedrine was administered. VAS, STAI-1, and STAI-2 values had a statistically significant effect on the occurrence of bradycardia (logistic regression analysis; *p*=0.01).

The mean age of the patients was 33.11 ± 12.2 (Table 3). While no significant correlation was found between age and motor and sensory block onset time, there was a statistically significant, positive, low-level correlation between age and time of motor block recovery (Spearman correlation analysis; *r* = 0.245; *p*=0.02).

VAS, STAI-1, STAI-2, sensory block onset, motor block onset, and motor block recovery time were higher in female patients (logistic regression analysis; *p*=0.002). Among patients, 70% (63) were males and 30% (27) were females. STAI-1 and STAI-2 and VAS values were mean 38.97 ± 9.9, 41.43 ± 8.7, and 5.24 ± 2.4 in males and mean 43.74 ± 8.9, 42.30 ± 7.0, and 7.15 ± 2.1 in females, respectively (Table 3). Sedation was given to 83 patients (92.22%) intraoperatively.

### 3.2. Discussion

The stress response to surgery occurs due to the release of catabolic hormones and increased plasma concentrations of catecholamines, cortisol, and corticotropin [12]. By blocking the afferent and efferent effects of the stress response to surgery and reducing the release of catecholamines with effective regional anesthesia, the surgical stress response is partially or completely suppressed [12, 13]. Anxiety also increases intraoperative catecholamine release, adrenocorticotropin release from the hypothalamus, cortisol level in blood, and activation of the sympathetic nervous system [14]. Reducing stress and anxiety during elective surgery will reduce organ dysfunctions and complications by reducing the neurohormonal response to surgery [15]. There are also various studies about the effect of preoperative anxiety on postoperative pain [16].

Although spinal anesthesia is very easy to apply, has the necessary muscle relaxation effect without affecting respiration, and is a low-cost procedure, it can increase the anxiety caused by the patient being awake during the procedure and reduce patient satisfaction. In patients receiving spinal anesthesia, the duration of spinal motor and sensory block onset and the time of recovery from the block are related to the characteristics of the local anesthetic and the area applied. In this study, the plan was to investigate the effect of preoperative anxiety on the duration of neuroaxial block in addition to these factors. We evaluated the effect of preoperative anxiety on the motor and sensory block in spinal anesthesia. It was observed that there was no effect on the sensory block onset time, the motor block onset time, and the motor block recovery time (Table 2).

In patients undergoing spinal anesthesia, hypotension develops secondary to decreased systemic vascular resistance and vasodilation caused by sympathetic blockade [13, 17]. Hypotension occurs as a result of the sympathetic block, the higher the level of spinal anesthesia is, the more likely is its occurrence [18]. In our study, higher level of block was associated with higher incidence of hypotension (Tables 4 and 5). Studies found that preoperative anxiety is effective in the development of intraoperative hypotension [8]. Although the VAS, STAI-1, and STAI-2 variables did not have a statistically significant effect on the occurrence of hypotension in our study (logistic regression analysis; *p* = 0.077), a 1-unit change in the STAI-1 score was statistically significant and caused a 0.93-fold increase in hypotension. Although not statistically significant, 1-unit change in the VAS and STAI-2 variables was 1.334 and 1.031 times more effective on the formation of hypotension, respectively. Although the change in VAS, STAI-1, and STAI-2 variables was not statistically significant, it was effective at 12% on the occurrence of hypotension (*R*^2^ = 0.12).

The development of bradycardia due to sympathetic blockade is a common complication in spinal anesthesia [19]. Our study supports the view that STAI-1, STAI-2, and VAS values are associated with the development of bradycardia, so there is a relationship between anxiety and the development of bradycardia.

There are different results in the literature about the relationship between age and preoperative anxiety level. While there are studies that found the preoperative anxiety scores of elderly patients to be lower than young and middle-aged patients [1, 16, 20], it was also found that anxiety levels were lower at younger ages [10, 21, 22]. The fact that patients over 55 years were not included in our study is thought to be a factor in the absence of a statistically significant relationship between age with VAS, STAI-1, and STAI-2 scores (Spearman correlation analysis; *p* = 0.390, *p* = 0.206, and *p* = 0.862, respectively) (Table 1).

It is known that women's preoperative anxiety levels are higher than men [23], and this was attributed to reasons such as separation from the family causing more anxiety in women than in men [24] and women's ability to express themselves better than men [1].

In our study, the variables of VAS, STAI-1, STAI-2, sensory block onset, motor block onset, and motor block recovery time were statistically significantly higher in women (logistic regression analysis; *p*=0.002).

Considering that reducing anxiety has effects on intraoperative catecholamine release, we can attribute the lack of a statistically significant difference in the times of motor block onset, sensory block onset, and recovery of motor block to the low number of patients not given sedation in our study. The amount of sedation given during the operation did not have an effect on the duration of the block (Spearman correlation analysis; *p*=0.929).

### 3.3. Limitations

Because the study was a prospective observational study, no randomization was made for patients who received and did not receive intraoperative sedation. This is an important limitation of our study. The number of patients with and without sedation was not equal. This may have caused statistical problems in assessing the effect of sedation on motor block onset, sensory block onset, and motor block recovery times.

## 4. Conclusions

Preoperative anxiety is an unpleasant feeling and may even be the reason why spinal anesthesia is not performed. High anxiety can impair preoperative patient satisfaction and service quality. It was found that preoperative anxiety in patients who underwent spinal anesthesia was associated with the development of bradycardia and, to a lesser extent, hypotension, but it had no effect on the time of onset of motor and sensory blocks and recovery from the motor block.

## Figures and Tables

**Table 1:** Patients' diagnoses.

	*n*	%
Pilonidal sinus	40	44.44
Inguinal hernia	21	23.33
Anal fistula	9	10
Benign soft tissue lesion	4	4.44
Ureter stone	3	3.33
Varicosis	2	2.22
Hemorrhoid	2	2.22
Varicocele	2	2.22
Condiloma	2	2.22
Scar revision	2	2.22
Others	3	3.36

**Table 2:** Relationship of all values with sensory block onset time, motor block onset time, and motor block recovery time.

	Sensory block onset time (*n* = 90)		Motor block onset time (*n* = 90)		Motor block recovery time (*n* = 90)	
	*r*	*p*	*r*	*p*	*r*	*p*
Age	0.86	0.422	0.079	0.458	0.181	0.088
Size	0.066	0.534	0.025	0.815	0.113	0.288
Weight	0.028	0.794	0.081	0.447	0.045	0.671
BMI	0.007	0.950	0.081	0.448	0.045	0.671
STAI-1	0.068	0.523	0.070	0.515	0.126	0.236
STAI-2	0.086	0.423	0.140	0.188	0.103	0.335
VAS	0.090	0.396	0.013	0.907	0.184	0.083
Operation time	0.127	0.233	0.069	0.521	0.530^∗∗^	<0.001
Total sedation	0.037	0.730	0.006	0.956	0.017	0.875

^∗∗^ *p* < 0.01.

**Table 3:** Descriptive statistics of patient demographic characteristics, anxiety scales, and other values.

	Mean ± SD	Min.–Max.
Age	33.11 ± 12.2	18–56
Size (cm)	171.53 ± 9	155–190
Weight (kg)	78.17 ± 13.7	47–118
BMI	26.61 ± 4.9	17.5–48.2
STAI-1	40.4 ± 9.8	20–63
STAI-2	41.69 ± 8.2	24–59
VAS	5.81 ± 2.5	0–10
Sensory block onset time (min)	0.89 ± 0.4	0.41–2
Motor block onset time (min)	5.81 ± 4	0–20
Operation time (min)	53.30 ± 27.9	12–150
Total sedation (midazolam/mg)	2.08 ± 1.1	0–6
Motor block recovery time (min)	92.06 ± 36.9	0–215

**Table 4:** Comparison of the highest sensory block level according to the presence of developing complications.

Highest sensory block level			
		Mean ± SD	*p*

Bradycardia	No *n* = 77	8.03 ± 1.6	0.134
	Yes *n* = 13	7.38 ± 1.8	

Atropine	No *n* = 77	8.03 ± 1.6	0.134
	Yes *n* = 13	7.38 ± 1.8	

Hypotension	No *n* = 74	8.12 ± 1.7	0.031^∗^
	Yes *n* = 16	7.06 ± 1.2	

Ephedrine	No *n* = 74	8.12 ± 1.7	0.031^∗^
	Yes *n* = 16	7.06 ± 1.2	

^∗^ *p* > 0.05; Mann–Whitney *U* test.

**Table 5:** Patients' sensory and motor levels.

	Level	*n*
Sensory level by the pinprick test	T6	26
	T7	18
	T8	9
	T9	12
	T10	23
	T11	2

Motor block by the Bromage scale	3	85
	2	3
	1	2
	0	0

## Data Availability

The datasets used and/or analysed during the current study are available from the corresponding author on reasonable request.
